# Clinical Issues in Elder Abuse Research

**DOI:** 10.1093/geroni/igaf122.1479

**Published:** 2025-12-31

**Authors:** Laura Mosqueda

**Affiliations:** University of Southern California, Pasadena, California, United States

## Abstract

There are several areas in the clinical arena that have come a long way in recent decades yet still have a long way to go. Medical forensic markers of abuse/neglect, i.e. those symptoms, signs, and data that may show evidence of a crime are notoriously difficult to distinguish from normal and common age-related changes of aging. While bruises and some types of fractures have been studied, other markers such as pressure sores and behavioral indicators deserve to be researched as well. Another important clinical aspect relates to going upstream, to the prevention of abuse/neglect. There are well-documented risk and associated factors for elder mistreatment as an umbrella term, and it is time to distinguish among the sub-types of abuse as we study causes and consequences. For example, it may be that the risk factors for neglect are quite different than those for physical abuse which would then have significant implications for prevention. As the field matures, we ought and now need to develop a more nuanced understanding and approach to our work and future research can help to achieve this. This brief presentation will consider what is known about this field (from past work), what is happening now (current work and initiatives) and what do we need to know (possible work in future).

